# Efficacy and Safety of Zhenyuan Capsule for Coronary Heart Disease with Abnormal Glucose and Lipid Metabolism: Study Protocol for a Randomized, Double-Blind, Parallel-Controlled, Multicenter Clinical Trial

**DOI:** 10.1155/2018/1716430

**Published:** 2018-04-26

**Authors:** Yu Qiao, Jingchun Zhang, Yue Liu, Zhiqi Liang, Yuhua Wang, Wei Zheng, Dazhuo Shi

**Affiliations:** ^1^Cardiovascular Diseases Center, Xiyuan Hospital, China Academy of Chinese Medical Sciences, 1 Xiyuan Caochang, Haidian District, Beijing 100091, China; ^2^Institute of Cardiovascular Diseases, China Academy of Chinese Medical Sciences, 1 Xiyuan Caochang, Haidian District, Beijing 100091, China; ^3^Jilin Jian Yisheng Pharmaceutical Co., Ltd., Ji'an, Jilin Province 134200, China

## Abstract

**Background:**

Coronary heart disease (CHD) and abnormal glucose and lipid metabolism are closely associated and generally coexist. The Qi and Yin deficiency syndrome is a common disease pattern encountered in traditional Chinese medicine. We designed a protocol to determine the effectiveness and safety of Zhenyuan capsules for CHD with abnormal glucose and lipid metabolism.

**Methods:**

This multicenter, randomized, double-blind, parallel-controlled trial was designed in accordance with the CONSORT. We will recruit 200 eligible male patients aged 45–75 years from three participating centers and randomly assign them to treatment and control groups (1 : 1). The primary indicators are glycosylated hemoglobin, fasting blood glucose, 2-hour postprandial blood glucose, and triglyceride levels. The secondary indicators are the Seattle Angina Questionnaire, TCM symptom indicators, ultrasonic cardiography finding, coagulation indicator, and P-selectin level. Measurements will be performed at baseline (T0), the end of the run-in period (T1), and weeks 4 (T2), 8 (T3), and 12 (T4) of the treatment period. Adverse events will be monitored during the trial.

**Discussion:**

This study aims to evaluate the efficacy and safety of Zhenyuan capsules in patients with CHD and abnormal glucose and lipid metabolism. The results will provide critical evidence of the usefulness of the Chinese herbal medicine for CHD with abnormal glucose and lipid metabolism.

**Trial Registration:**

This trial is registered with the Chinese Clinical Trials Registry, with identifier number ChiCTR-TRC-14004639, May 4, 2014.

## 1. Introduction

Treatment of coronary heart disease (CHD), which poses a significant threat worldwide, has made great progress, including thrombolysis, intervention, and coronary artery bypass grafting. However, according to the COURAGE study, these interventional therapies may not effectively reduce the risk of major adverse cardiac events in patients with stable CHD as compared with conservative treatment [1]. Therefore, strengthening drug treatment, controlling cardiovascular risk factors, and lifestyle modification intervention on smoking and exercise are considered the most basic measures.

In addition, diabetes mellitus as an important independent risk factor of cardiovascular disease has been described in the 1999 American Heart Association statement as “a cardiovascular disease” and regarded as a coronary disease equivalent by the National Cholesterol Education Program (NCEP) Adult Treatment Panel III 2001 [2]. In fact, following the growth of living standard, the incidence of type 2 diabetes is increasing yearly. In 2007-2008, the prevalence rates of total diabetes and prediabetes were 9.7% (approximately 92.4 million adults) and 15.5% (approximately 148.2 million adults), respectively [3]. The risk of macroangiopathy increases significantly in diabetes even during the prediabetic state. Fortunately, the 10-year follow-up results of the UK Prospective Diabetes Study showed that patients with intensive glucose control initially showed risk reduction for any diabetes-related end-point by 9% (*P* = 0.04), microvascular disease by 24% (*P* = 0.001), myocardial infarction by 15% (*P* = 0.01), and all-cause mortality by 13% (*P* = 0.007). Moreover, myocardial infarction or all-cause mortality risk was decreased significantly [4]. Thus, early detection and intervention in diabetes are important measures to reduce the morbidity of myocardial infarction and decrease mortality in CHD patients. Glycosylated hemoglobin (HbA1c) level, a test used to monitor the effectiveness of diabetes treatment, is closely related to vascular endothelial injury and coronary atherosclerosis. By measuring HbA1c level, abnormal glucose metabolism can be detected, and then the incidence of CHD can be prevented by controlling glucose levels.

With the publication of the Scandinavian Simvastatin Survival Study, the Cholesterol and Recurrent Events, and other large-scale clinical trials, the relationship between blood lipid levels and cardiovascular atherosclerosis has gradually become clear. Dyslipidemia is closely related to atherosclerosis and is an independent risk factor of CHD. Previous studies showed that lipid lowering can significantly reduce the risk of CHD by 2%, with total cholesterol and low-density lipoprotein cholesterol (LDL-C) both lowered by 1%. Extremely low levels of high-density lipoprotein cholesterol have been shown to increase the risk of CHD by 70% in men and 100% in women. Therefore, controlling abnormalities in serum lipid levels is an important part of the secondary prevention of CHD. The morbidity and mortality of CHD could be significantly reduced by using lipid-lowering drugs, which also could slow down and reverse the progression of atherosclerosis.

Furthermore, dyslipidemia, which is associated with diabetes, is the main cause and one of the major risk factors of diabetes-associated macrovascular complications [5]. Owing to the abnormally elevated lipids deposited in the cell and vessel wall by some mechanisms, atherosclerotic plaques are formed, and, finally, stenosis, even occlusion, occurs in the blood vessel lumen. According to NCEP, diabetes has a high risk of developing into CHD within 10 years. The primary preventive goal is to reduce the LDL-C level [6]. Moreover, CHD with diabetes may occur with a more serious lipid metabolism disorder. For these reasons, more attention should be paid to the treatment of dyslipidemia and diabetes in CHD patients.

Traditional Chinese medicine (TCM) emphasizes symptomatic treatment and has long-term application in CHD-related diseases. From the view of TCM, patients with CHD and abnormal glucose and lipid metabolism can be divided according to symptoms. A common symptom is the Qi and Yin deficiency syndrome. The effect of Zhenyuan capsules in the treatment of this type of patients has been observed in the clinical setting. The main component of the Zhenyuan capsule is ginseng fruit saponin (GFS), a ginsenoside extract from the mature fruit of the* Panax ginseng *fruit. Previous studies showed multiple components of saponin in GFS, such as ginsenosides Re, Rb1, Rb2, Rc, Rg1, Rg2, and Rd. The structures of these compounds are identified on the basis of physicochemical constants, ultraviolet light, nuclear magnetic resonance, and spectral analysis. The study by Xu et al. [7] showed that the main component of GFS is ginsenoside Re, which accounts for as much as 85% of the GFS content, 30 times higher than the content in ginseng roots and 6% in all ginseng. The remainder is a small amount of ginsenoside Rg2 and trace amounts of ginsenoside Rg1. This means that ginsenoside Re may be the main active constituent. In addition, modern pharmacological studies have shown that GFS has the effect of lowering blood pressure and blood glucose level, countering arrhythmias, protecting against myocardial ischemia-reperfusion injury, and other actions.

Therefore, we designed this randomized, double-blind, parallel-controlled, multicenter, phase IV clinical trial to investigate the efficacy of the Zhenyuan capsule in the treatment of CHD accompanied with glucose and lipid metabolic disorders. The outcome measures are focused on symptom reduction and improvement in physiochemical indexes. We will also determine clinical safety.

## 2. Methods/Design

### 2.1. Study Design

This study is registered with the Chinese Clinical Trial Registry (registration number ChiCTR-TRC-14004639). The multicenter, randomized, double-blind, parallel-controlled, phase IV clinical study follows the recommendations of the CONSORT [8] and SPIRIT statements [9]. This study is financially supported by Jilin Jian Yisheng Pharmaceutical Co. Ltd., Jilin, China. The company is not involved in the clinical trial design, data collection and analysis, or other parts of the study.

**Figure 1:**
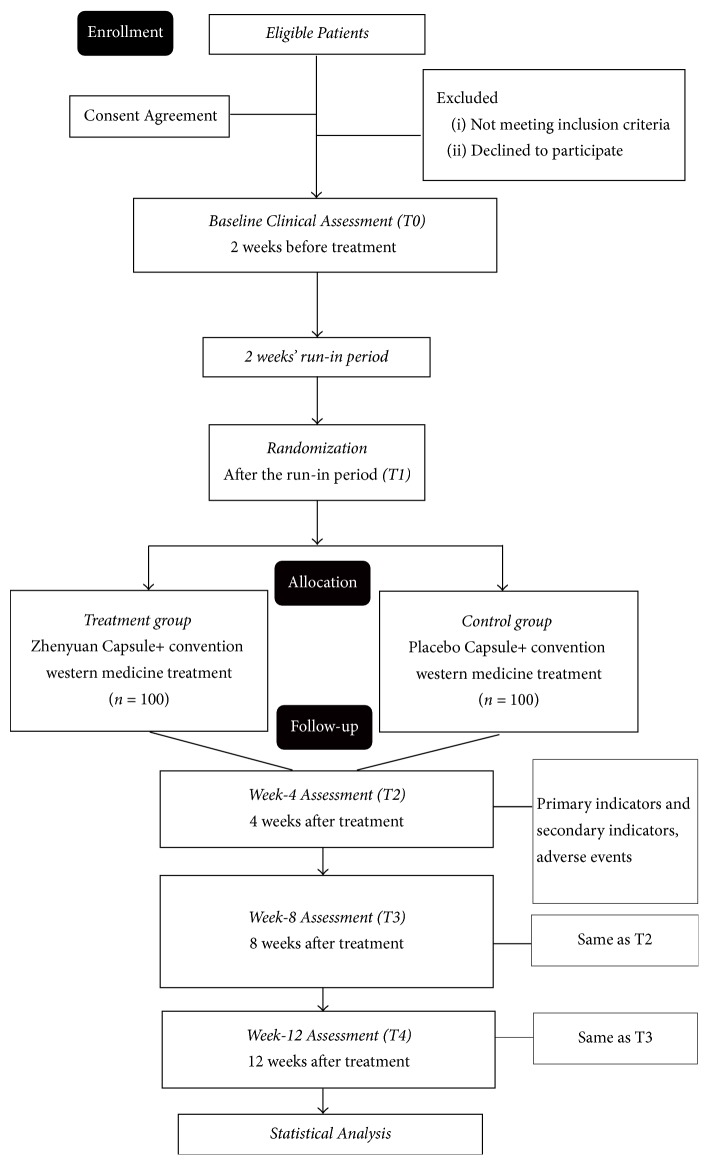
Study flowchart.

Participants will be recruited from three participating centers, namely, Xiyuan Hospital of China Academy of Chinese Medical Sciences, Guan'anmen Hospital of China Academy of Chinese Medical Sciences, and the Second Affiliated Hospital of Tianjin University of TCM. The leading unit among the three centers is Xiyuan Hospital. In this trial, 200 patients will be recruited and randomly allocated to two groups (treatment group, *n* = 100; control group, *n* = 100). Clinical assessment will be performed at baseline (T0, 2 weeks preceding the treatment) and at the end of the 2-week run-in period (T1). Participants will then receive the treatment for 12 weeks. In the treatment period, the intervention assessments are performed at weeks 4 (T2), 8 (T3), and 12 (T4).  Figure 1 shows the study procedures.

### 2.2. Participants

Inpatients and outpatients will be screened and identified in three participating centers strictly according to the inclusion and exclusion criteria. Written informed consent of each participating patient is required for entry to the study.

#### 2.2.1. Inclusion Criteria

Subjects are eligible for study participation if they meet the following criteria: (1) coronary angiography or coronary computed tomographic angiography shows that at least one of the main branches of the coronary artery has stenosis of at least 50%; (2) TG levels between 1.70 and 2.25 mmol/L and HbA1c level between 5.7% and 7%; (3) the TCM syndrome differentiation is Qi and Yin deficiency; (4) age between 45 and 75 years at screening; and (5) providing informed consent form.

#### 2.2.2. Exclusion Criteria

Participants will be excluded if they report any of the following criteria: (1) serious cardiovascular diseases, including severe valvular heart disease, heart valve replacement, refractory heart failure, cardiac function grade IV (NYHA), and cardiogenic shock or serious cardiac insufficiency (EF < 35%); (2) mental disease or malignant tumor; (3) renal insufficiency, serum creatinine level of >2.5 mg/dl for men (>220 *μ*mol/l) and >2.0 mg/dl (>175 *μ*mol/l) for women; (4) hypohepatia, liver enzyme level of >2 times the normal value; (5) high risks of hemorrhage, hemophilia, and platelet dysfunction, or history of thrombocytopenia, which might influence the use of antiplatelet drugs; (6) active or emerging (<3 months) bleeding, including gastrointestinal bleeding, stool occult blood, or gross hematuria; (7) pregnant or attempting to become pregnant; (8) allergic constitution; (9) joined another trial or received random allocation in another study within the past 3 months; or (10) being unable or unwilling to sign the informed consent form, being noncompliant, or having poor possibility of a follow-up visit.

#### 2.2.3. Withdrawal, Dropout, and Discontinuation

Participation will be discontinued if the participant has sudden worsening of condition that is not confirmed to be associated with this trial, or serious adverse events, or is unable to cooperate with the trial investigators and/or to complete the follow-up. If the condition continues to worsen, complications or special physiological changes emerge, a participant refuses to continue the trial, or a participant uses medicines that are not permitted in this trial, the patient will be withdrawn from the trial after the researcher's assessment. The data will be considered invalid but will still be included in the full analysis set (FAS). Information from dropouts will be collected by phone interviews, such as the reason for the trial discontinuation and the status of the final medication received. Participants who withdraw from the trial will receive appropriate treatment for adverse events.

### 2.3. Intervention

Patients who meet all the inclusion and none of the exclusion criteria will be randomized into two groups as follows: (1) treatment group, 0.5 g of Zhenyuan capsule, 3 times/day, and (2) control group, 0.5 g of placebo, 3 times/day. According to the Guideline for the Diagnosis and Treatment of Chronic Stable Angina, participants can maintain the conventional drug treatments such as antiplatelet agents, statins, nitrates, anticoagulation drugs, *β*-blockers, calcium channel antagonists, angiotensin-converting enzyme inhibitors, or angiotensin II receptor antagonists. However, statins are the only Western medicine for lipid treatment. Patients diagnosed as having type 2 diabetes maintain the current Western medicines for hypoglycemic effects. To avoid discussing their medications among themselves, participants are advised to take the drugs at home and required to remain isolated from each other. In addition, the treatment allocation remains blind throughout the study. Participants will not be permitted to use other treatments for angina pectoris in CHD and diabetes, including TCM herbs and Chinese patent medicine. In the run-in period, any TCM medications for improving blood lipid or glucose levels will not be permitted.

### 2.4. Outcomes and Measures

The primary indicators, HbA1c, fasting blood glucose (FBG), 2-hour postprandial blood glucose (2hPBG), and triglyceride (TG) levels, will be tested at T0, T1, T2, T3, and T4. The secondary indicators, ultrasonic cardiography (UCG) finding, coagulation indicator, and P- selectin level, will be assessed at T1 and T4. Cardiac death, nonfatal myocardial infarction, coronary revascularization, Seattle Angina Questionnaire (SAQ) score, and TCM symptom indicators will also be monitored periodically. Adverse events, which include hemorrhage, will be followed up using electrocardiography, routine urinalysis, routine stool test, and liver and kidney function tests (ALT, AST, Cr, and BUN) from T1 to T4. Items to be measured and the time window of data collection are shown in Table 1.

### 2.5. Adverse Events

Participants are required to honestly report any changes in their symptoms. Any adverse events or unexpected toxic side effects that develop during the trial will be analyzed to determine the cause. These will be recorded and treated appropriately by the intervention administrators. In cases of severe adverse events, the participating patients are asked to take immediate measures to protect their safety and report the events to the Drug Supervision Bureau, the trial applicant, and the ethics committee within 24 hours.

### 2.6. Study-Specific Visits and Procedures

Table 1 shows the study-related procedures. All eligible patients should have a 2-week run-in period followed by the treatment period. Participants are assessed by the same researcher consistently throughout the trial. Baseline measures include demographic data; medical and treatment history; medications; vital signs (temperature, breathing, heart rate, and blood pressure); FBG, 2hPDG, HbA1c, and TG levels; and liver and kidney function tests. Baseline measurements will be repeated with all participants at T1 and T4. The TCM syndrome indicators, SAQ, adverse events, and outcome measures will be recorded from T1 to T4.

### 2.7. Randomization and Blinding

Eligible patients are randomly allocated to the treatment or control group in a 1 : 1 ratio. The randomization sequence will be recorded by staff at the sponsor unit. The researchers and outcome assessors participating in this study have no knowledge of the randomization sequence details. In addition, the results are kept in sealed, opaque, and stapled envelopes. They will be unsealed at the end of the outcome assessment. Emergency letters, including random codes and group assignments, are prepared by a statistician. If the blind code is exposed, patients will be excluded accordingly. Any of the following situations could call for a blind breaking and urgent measure: a serious adverse event, serious complication, or a deterioration in the patient's condition.

### 2.8. Data Entry and Quality Control

The data of the participants collected according to the Case Report Forms will be imported into the clinical data management system (http://www.xyedc.com/). To keep the data consistency, the supervisor will compare the electronic database with the source documents and correct any identified errors. In addition, computer logic checks will be run after data entry. Manual checks and the Data Coordination Center will also help to identify more complicated and less common errors. All these measures are used to ensure the accuracy and integrity of information.

### 2.9. Sample Size Calculation

The sample size was determined with a type I error rate of *α* = 0.05 and a power of 80% (type II error rate of *β* = 0.2). According to a previous study, the efficacy rate of Zhenyuan capsules is 94% for relieving angina pectoris symptoms and 64% for placebo [10]. The sample size should therefore consist of 25 participants in each group. Considering the potential 20% loss and the cases that can be counted in each center, 100 participants are needed for each group. As treatment and control groups, and three centers are involved, 200 participants will be recruited in this study. The formula used to calculate the sample size is as follows:

(1) $$\begin{array}{c} n=\frac{p_{1}\left(1-p_{1}\right)+p_{2}\left(1-p_{2}\right)}{{\left(p_{1}-p_{2}\right)}^{2}}{\left(\;u_{1-\alpha }+u_{1-\beta }\;\right)}^{2}, \end{array}$$

*n* ≈ 25 patients/group.

### 2.10. Statistical Analyses

Statistical analyses will be conducted by an independent statistician from Xiyuan Hospital. In the FAS, patients will be dosed with the study drugs at least one time and one clinical observation will be recorded. Participants with good compliance and without any protocol deviations will be included in the per-protocol set (PPS). Subjects with safety data who have completed at least one study visit will be included in the safety analysis. The mean ± SD will be used in continuous variables. The characteristic comparability between the two groups will be evaluated by a two-sample Student *t*-test for continuous variables and the Fisher exact test or *χ*^2^ test for categorical variables. In this multicenter trial, the Cochran-Mantel-Haenszel test and the analysis of variance will be used. For diminishing the influence of confounding factors on the curative effect, analysis of covariance and the logistic regression model will be applied. For all the analyses, the difference with a value of *P* < 0.05 will be statistically significant. All analyses will be conducted using the SAS software version 9.3 (SAS Institute, Cary, NC, USA).

## 3. Discussion

Recently, many studies have used the Zhenyuan capsule as treatment for CHD, heart failure [11], and type 2 diabetes [12] with Qi deficiency or blood stasis. The benefits of the treatment are clear. In addition, the effect of the Zhenyuan capsule in the treatment of CHD with abnormal glucose and lipid metabolism has been observed in clinical settings, but the evidence is not strong. This study intends to examine the clinical efficacy and safety of the Zhenyuan capsule for treating these diseases in a randomized, double- blind, and parallel-controlled trial.

This study first applies the Zhenyuan capsule for blood lipid research. This may reveal new uses for old drugs in TCM. In addition, this is the first study to address the efficacy and safety of the Zhenyuan capsule for CHD with abnormal glucose and lipid metabolism in a double-blind and parallel-controlled trial. We assume that this design, which is in line with the TCM theory, will contribute to more convincing results and robust evidence for the efficacy of study medications.

This study has some limitations. Owing to the complexity of the disease, many potential influencing factors may exist. Therefore, to minimize possible errors, participants will have a 2-week run-in period. During this period, any TCM medications for improving blood lipids or glucose will not be permitted. In addition, participants will not be permitted to use other treatments for angina pectoris in CHD and diabetes mellitus, including TCM herbs and Chinese patent medicines at any time during the study.

In summary, by rigorous design, the results of this study will provide clinical evidence regarding the efficacy and safety of Zhenyuan capsules for CHD with abnormal glucose and lipid metabolism.

## Figures and Tables

**Table 1:** Measurement items and time points for data collection.

	Screening	Treatment period			
	T0	Run-in period (T1)	Week 4 (T2)	Week 8 (T3)	Week 12 (T4)
*Basic medical history recording*					
Informed consent	×				
Inclusion/exclusion criteria		×			
Demographic data		×			
Medical and treatment history recording		×			
Complication and symptom recording		×			
Concomitant medication		×	×	×	×
Vital signs		×	×	×	×
*Efficacy observation*					
TG, FBG, 2hPBG, and HbA1c levels	×	×	×	×	×
TCM symptom indicators		×	×	×	×
Seattle Angina Questionnaire score		×	×	×	×
Coagulation indicator		×	×	×	×
P-selectin level		×	×	×	×
Angina pectoris scores and end-point event		×	×	×	×
ECG		×			×
UCG		×			×
*Safety assessment*					
Liver and renal function tests	×	×	×	×	×
Hemorrhage		×	×	×	×
Routine blood and urine tests		×			×
Routine stool and occult blood tests		×			×
*Other works*					
Randomization and allocation		×			
Drug distribution		×	×	×	
Medicine recycling and drug quantity statistics			×	×	×

## Abbreviations

ALT: Alanine transferase
BUN: Blood urine nitrogen
CHD: Coronary heart disease
COURAGE: The Clinical Outcomes Utilizing Revascularization and Aggressive Drug Evaluation
CR: Serum creatinine
CRF: Case report form
CTA: Computed tomographic angiography
ECG: Electrocardiography
FAS: Full analysis set
FBG: Fasting blood glucose
GFS: Ginseng fruit saponin
HbA1c: Glycosylated hemoglobin
LDL-C: Low-density lipoprotein cholesterol
NCEP: National Cholesterol Education Program
PPS: Per-protocol set
SAQ: Seattle Angina Questionnaire
TC: Total cholesterol
TCM: Traditional Chinese medicine
UCG: Ultrasonic cardiography
2hPBG: 2-hour postprandial blood glucose.

## Additional Points

*Trial Status*. The trial was initiated in May 2014 and is currently open for enrollment. No analysis has been conducted since the commencement of the trial.

## References

[1] Boden W., O’Rourke R., Teo K. (2007). Optimal medical therapy with or without PCI for stable coronary disease. *The New England Journal of Medicine*.

[2] American Medical Association (2001). Executive summary of the third report of the National Cholesterol Education Program (NCEP) expert panel on detection, evaluation, and treatment of high blood cholesterol in adults (adult treatment panel III). *Journal of the American Medical Association*.

[3] Yang W., Lu J., Weng J. (2010). Prevalence of diabetes among men and women in China. *The New England Journal of Medicine*.

[4] Holman R. R., Paul S. K., Bethel M. A., Matthews D. R., Neil H. A. W. (2008). 10-Year follow-up of intensive glucose control in type 2 diabetes. *The New England Journal of Medicine*.

[5] Rauchhaus M., Coats A. J. S., Anker S. D. (2000). The endotoxin-lipoprotein hypothesis. *The Lancet*.

[6] Walter D. H., Schächinger V., Elsner M., Mach S., Auch-Schwelk W., Zeiher A. M. (2000). Effect of statin therapy on restenosis after coronary stent implantation. *American Journal of Cardiology*.

[7] Xu C., Cui S., Zheng Y. (2004). Determination of the amount of ginsenosides-Rg1, -Re, -Rb1, -Rc, - Rb2 and -Rd in leaves, stems and fruit extraction of pannax ginseng by RP-HPLC. *Journal of Jilin Agricultural University*.

[8] Moher D., Hopewell S., Schulz K. F. (2012). CONSORT 2010 explanation and elaboration: updated guidelines for reporting parallel group randomised trials. *International Journal of Surgery*.

[9] Chan A. W., Tetzlaff J. M., Altman D. G. (2013). SPIRIT 2013 statement: defining standard protocol items for clinical trials. *Annals of Internal Medicine*.

[10] Zeng D., Qi X., Zheng X. (2001). Clinical observation of Zhenyuan capsule in the treatment of coronary heart disease and angina pectoris. *Chinese Journal of Cardiology*.

[11] Cao Y., Wang W., Lu L. (2017). Adjuvant effects of zhenyuan capsule on the cardiac function of patients with chronic heart failure: a meta-analysis. *China Journal of Chinese Materia Medica*.

[12] Liu Q. (2011). Zhenyuan Capsule Insulin Treatment of type2 Diabetes 32 cases of Angina Pectoris. *Journal of Practical Traditional Chinese Internal Medicine*.

